# Oil-Based Sanitization in Low-Moisture Environments: Delivery of Acetic Acid with Water-in-Oil Emulsions

**DOI:** 10.1128/spectrum.05293-22

**Published:** 2023-04-05

**Authors:** Shihyu Chuang, Mrinalini Ghoshal, Lynne McLandsborough

**Affiliations:** a Department of Food Science, University of Massachusetts—Amherst, Amherst, Massachusetts, USA; b Department of Microbiology, University of Massachusetts—Amherst, Amherst, Massachusetts, USA; Oregon State University

**Keywords:** cleaning and sanitation, desiccated bacteria, dry food processing, oil-based antimicrobial delivery, osmotic pressure, water-in-oil emulsion, *Listeria monocytogenes*, low-moisture foods, oil-based delivery, organic acids, *Salmonella enterica*

## Abstract

Contamination with Salmonella spp. and Listeria monocytogenes is concerning across low-moisture food (LMF)-processing environments due to the pronounced survival of these organisms under dry conditions. This study treated desiccated bacteria with acetic acid delivered by oil with and without water-in-oil (W/O) emulsion. The influences of cellular desiccation, emulsion water concentration, water activity (*a_w_*), and treatment temperature were investigated. Acetic acid dissolved in oil (i.e., acidified oil) showed low levels of antimicrobial efficacy. After treatment with acidified oil (200 mM acetic acid at 22°C for 30 min), Salmonella enterica serovar Enteritidis phage type 30 cells desiccated to 75% equilibrium relative humidity (ERH) and 33% ERH were reduced by 0.69 and 0.05 log CFU/coupon, respectively. The dispersion of a low level of water (≥0.3%, vol/vol) within the acidified oil with the surfactant (i.e., acidified W/O emulsion) significantly enhanced the antimicrobial efficacy. After treatment with the acidified W/O emulsion (200 mM acetic acid at 22°C for 20 min), desiccated Salmonella (4-strain cocktail) and L. monocytogenes (3-strain cocktail) cells were reduced by >6.52 log most probable number (MPN)/coupon, regardless of the desiccation levels. Increased efficacy was observed with temperature elevation. Reduced efficacy was observed when glycerol was added to the aqueous phase of the emulsion to decrease the solution *a_w_*, indicating that the enhanced efficacy of the acidified W/O emulsion was associated with differential osmotic pressure. The antimicrobial mechanism may be due to the membrane disruption induced by acetic acid, in combination with the hypoosmotic stress provided by W/O emulsion, creating cellular lysis, as illustrated by electron micrographs.

**IMPORTANCE** Aqueous-based cleaning and sanitation are undesirable in processing facilities that manufacture low-moisture foods such as peanut butter and chocolate. Alcohol-based sanitization is advantageous because it leaves no residue on the contact surface but requires the processing facility to close temporarily due to flammability. At >6.52 log kill of desiccated Salmonella and Listeria monocytogenes cells, the developed oil-based formulation has the potential to be an effective dry sanitation method.

## INTRODUCTION

Low-moisture foods (LMFs) are foods low in moisture content with a water activity (*a_w_*) of less than 0.85, which does not support the growth of microorganisms (1). However, it has been reported that some bacterial pathogens such as Salmonella spp. and Listeria monocytogenes can survive within food matrices for prolonged periods (2, 3). Salmonella contamination is of particular concern in LMFs such as peanut butter and chocolate as this organism exhibits increased resistance to heat in low-*a_w_* environments (4–8), which is thought to be a factor contributing to numerous foodborne outbreaks (9–17). Although no L. monocytogenes outbreaks have been associated with LMFs, some products containing peanut butter and chocolate have been recalled due to the presence of this organism (18). In addition, contamination with Salmonella and L. monocytogenes poses a risk to dry pet food manufacturing (19). Household animals consuming contaminated products can become a source of human infections.

A sanitation protocol based on water is undesirable in LMF-processing environments due to the immiscible nature of water and lipids, and the presence of water residing on the equipment surface can lead to microbial growth (20). One commercial dry cleaning method applies heated oil to flush out food debris, followed by alcohol-based sanitization (21). Alcohol-based sanitizers are advantageous in that no residue will be left on the applied surface. However, the processing facility must temporarily close for equipment cooldown after the hot oil rinse as alcohol is flammable, causing production downtime. Thus, developing sanitizers utilizing a heat-stable solvent such as oil would allow the increased frequency and efficiency of cleaning and sanitation.

Organic acids are naturally occurring substances with antimicrobial activity (22). It has been reported that desiccated Salmonella acquires higher tolerance to multiple environmental stresses and antimicrobial treatments except for exposure to organic acid solutions (23). Likewise, the efficacy of vaporized organic acids against desiccated Salmonella varies with the environmental relative humidity (24). Many organic acids are miscible with oil in the undissociated form and thus possess the potential for the development of oil-based sanitizers. Previously, we showed that the oil-based delivery of organic acids was a viable strategy against desiccated Salmonella (25). Acetic acid was the most effective food-grade acid, exhibiting a synergistic effect with heat. However, the efficacy was found to be contingent on the level of cellular desiccation, indicating that the levels of water and osmotic pressure may contribute to the antimicrobial activity of organic acids.

A water-in-oil (W/O) emulsion is a colloidal dispersion of water within a continuous oil phase. As emulsions are thermodynamically unstable and prone to breakdown over time via physiochemical mechanisms, it is essential to include stabilizers in the formulation for long-term stability (26). Emulsifiers/surfactants are some of the most critical stabilizers for food applications. These compounds are amphiphilic molecules that readily adsorb to the liquid-liquid interface and decrease the interfacial tension upon droplet formation, stabilizing the system (27).

This study aimed to investigate the efficacy of acetic acid against desiccated bacteria using a W/O emulsion as the delivery system. One Gram-negative and one Gram-positive system were used for the justification of antimicrobial testing.

## RESULTS

### Development of antimicrobial W/O emulsions.

The initial screening of acidified W/O emulsions was performed using Span 80 as the surfactant with spontaneous emulsification, which was found to have high antimicrobial efficacy. However, before moving forward, a shelf-life study was performed to compare the effects of different surfactants on stabilizing the emulsion (see Fig. S1 in the supplemental material). A difference in stability against gravitational separation was observed between the coarse acidified W/O emulsions that Span 80 and polyglycerol polyricinoleate (PGPR) stabilized. After storage at 22°C for 14 days, the PGPR-stabilized emulsion remained stable, with only a thin layer of oil rising to the top surface. Such oil-phase separation was more pronounced in the Span 80-stabilized counterpart, in addition to clear water-phase separation indicating droplet breakdown. This was in line with existing literature investigating the stabilization mechanisms of W/O emulsions with different surfactants (28). Thus, PGPR was selected for use as the surfactant in subsequent experiments.

### Antimicrobial efficacy of acidified oil and W/O emulsions.

**FIG 1:**
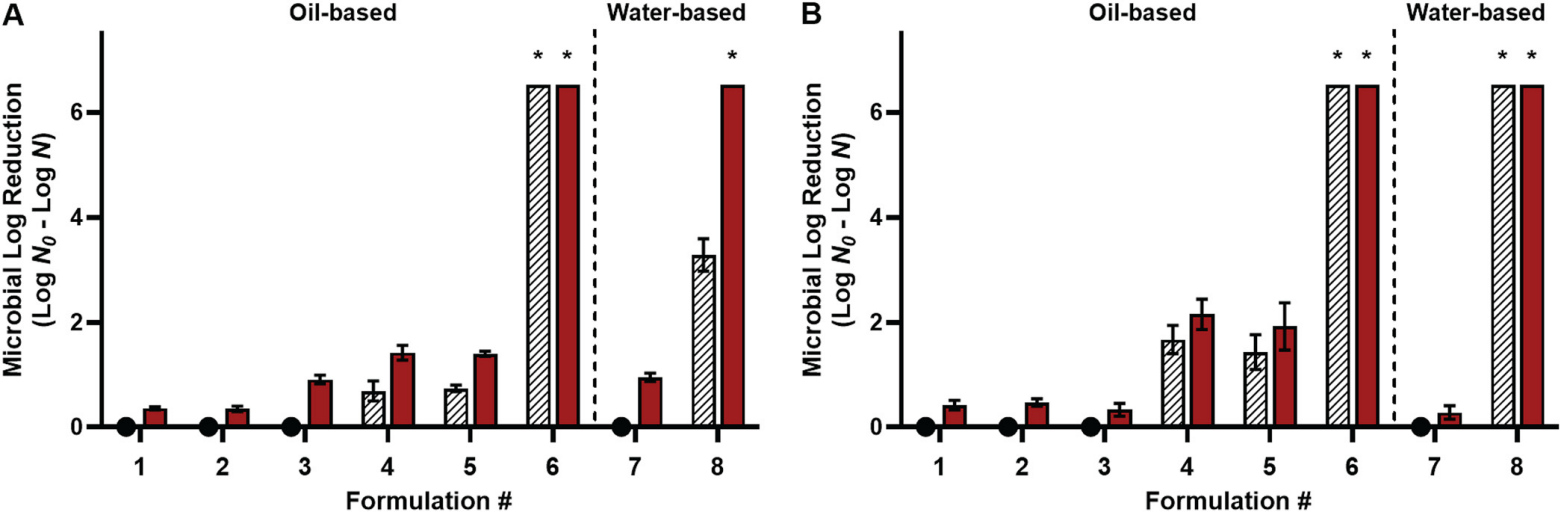
Efficacies of various treatment formulations at 22°C (striped white bars) and 45°C (filled red bars) against desiccated Salmonella Enteritidis phage type 30 (A) and Listeria monocytogenes LM25 (B). All treatment formulations were tested against cells desiccated to 75% ERH with a 30-min treatment time. The formulations included pure peanut oil (formulation 1); peanut oil with 3% (wt/wt) PGPR (formulation 2); peanut oil with 3% (wt/wt) PGPR and 3% (vol/vol) distilled water in the form of a water-in-oil (W/O) emulsion (formulation 3); peanut oil with 200 mM acetic acid (formulation 4); peanut oil with 200 mM acetic acid and 3% (wt/wt) PGPR (formulation 5); peanut oil with 200 mM acetic acid, 3% (wt/wt) PGPR, and 3% (vol/vol) distilled water as a W/O emulsion (formulation 6); distilled water (formulation 7); and distilled water with 200 mM acetic acid (formulation 8). The inoculum level was 7 log CFU per stainless steel coupon, and the detection limit was 0.48 log MPN. Nonacidified treatments at 22°C did not result in MLRs and thus are marked with closed circles with the bars omitted from the graph. *, bacterial survival was reduced to below the detectable limit, i.e., a reduction of >6.52 log MPN/coupon.

The oil-based delivery of acetic acid was tested against one strain of each organism for efficient screening, i.e., Salmonella enterica serovar Enteritidis phage type 30 and L. monocytogenes LM25. A 30-min contact time with 200 mM acetic acid was used against cells desiccated to 75% equilibrium relative humidity (ERH). Nonacidified controls included oil alone, oil with the surfactant, W/O emulsion (with 3% [vol/vol] distilled water), and water alone (Fig. 1). These treatments did not affect the desiccated bacteria at 22°C but resulted in microbial log reductions (MLRs) of <1 log CFU/coupon at 45°C. With acidified oil, the presence of the surfactant did not affect the antimicrobial efficacy (*P > *0.05). At 22°C, acidified oils with 200 mM acetic acid with and without PGPR reduced desiccated S. Enteritidis by 0.69 and 0.74 log CFU/coupon, respectively, and reduced desiccated L. monocytogenes by 1.67 and 1.43 log CFU/coupon, respectively. At 45°C, acidified oils with 200 mM acetic acid with and without PGPR reduced desiccated S. Enteritidis by 1.42 and 1.40 log CFU/coupon, respectively, and reduced desiccated L. monocytogenes by 2.15 and 1.92 log CFU/coupon, respectively. However, as 3% water was dispersed within the acidified oil with the surfactant to create acidified W/O emulsions as the treatment, both S. Enteritidis and L. monocytogenes numbers were reduced to below the detection limit of 0.48 log most probable number (MPN)/coupon (i.e., a reduction of >6.52 log MPN/coupon), with and without heating.

For comparison, acetic acid dissolved in water was tested. Acetic acid in water (200 mM) reduced desiccated S. Enteritidis by 3.28 log CFU/coupon at 22°C, which increased to >6.52 log MPN/coupon at 45°C. At both temperatures, desiccated L. monocytogenes was reduced by >6.52 log MPN/coupon. Without acetic acid, desiccated S. Enteritidis was more sensitive to water heating than oil (*P < *0.05). However, the dispersion of 3% water within the oil offset such a difference. These dry- and wet-heat phenomena were not observed for desiccated L. monocytogenes.

**FIG 2:**
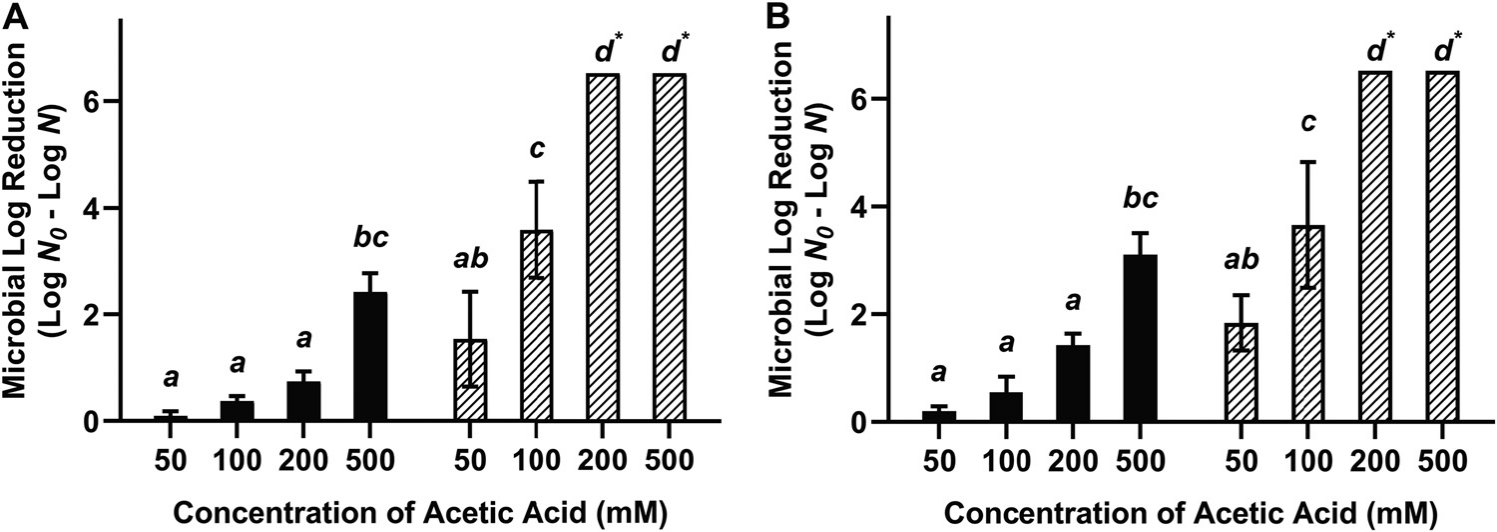
Influence of the acetic acid concentration on the efficacy of acidified oil (filled black bars) and acidified W/O emulsions (striped white bars) against desiccated Salmonella Enteritidis phage type 30 (A) and Listeria monocytogenes LM25 (B). All treatment formulations were tested against cells desiccated to 75% ERH with a 30-min treatment time at 22°C. The aqueous phase in acidified W/O emulsions was 3% (vol/vol) distilled water. The inoculum level was 7 log CFU per stainless steel coupon, and the detection limit was 0.48 log MPN. Different lowercase letters indicate statistical significance (*P < *0.05). *, bacterial survival was reduced to below the detectable limit, i.e., a reduction of >6.52 log MPN/coupon.

The influence of the acetic acid concentration on the antimicrobial efficacy of acidified oil and acidified W/O emulsions (3% water) was investigated (Fig. 2). A 30-min contact time at 22°C was used against cells desiccated to 75% ERH. Acidified oils with 50, 100, 200, and 500 mM acetic acid reduced desiccated S. Enteritidis by 0.10, 0.38, 0.74, and 2.41 log CFU/coupon, respectively (Fig. 2A, black bars), and reduced desiccated L. monocytogenes by 0.20, 0.55, 1.43, and 3.11 log CFU/coupon, respectively (Fig. 2B, black bars). Acidified W/O emulsions with 50 and 100 mM acetic acid reduced desiccated S. Enteritidis by 1.53 and 3.59 log CFU/coupon, respectively, which increased to >6.52 log MPN/coupon with ≥200 mM acetic acid (Fig. 2A, striped bars). Similar results were observed when L. monocytogenes was treated with acidified W/O emulsions, with acetic acid levels of 50 and 100 mM reducing the numbers of desiccated L. monocytogenes cells by 1.84 and 3.66 log CFU/coupon, respectively, which increased to >6.52 log MPN/coupon with ≥200 mM acetic acid (Fig. 2B, striped bars). The influence of the acetic acid concentration was statistically significant by analysis of variance (ANOVA) (*P < *0.05). The difference between acidified oil and acidified W/O emulsions was also statistically significant at each acetic acid concentration. Thus, 200 mM acetic acid in W/O emulsions was selected for subsequent experiments.

### Influence of droplet size on the antimicrobial efficacy of acidified W/O emulsions.

The coarse PGPR-stabilized acidified W/O emulsion presented a monomodal distribution of 1.2 μm (polydispersity index [PDI] of 0.23) upon formation, which shifted to a multimodal distribution averaging 10 μm (PDI of 0.66) after storage at 22°C for 14 days (Table 1). In comparison, the size of the fine PGPR-stabilized acidified W/O emulsion was 0.5 μm (PDI of 0.08), which did not show significant changes throughout the study, in agreement with the existing literature (29). The antimicrobial efficacy of acidified W/O emulsions (measured with a 30-min treatment at 22°C with 200 mM acetic acid against cells desiccated to 75% ERH) was not dependent on the particle size (Table 1). In addition, the tested carrier oils and surfactants did not change the antimicrobial efficacy (results not shown). Thus, the coarse PGPR-stabilized acidified W/O emulsion was selected for subsequent experiments.

**TABLE 1:** Dynamic light scattering analysis of the PGPR-stabilized acidified W/O emulsions (200 mM acetic acid, 3% [vol/vol] water, 3% [wt/wt] PGPR) over storage at 22°C for 14 days

Emulsification method^*a*^	Storage duration (days)	Mean particle diam (Z avg) (μm) ± SD	Distribution intensity (PDI)^*b*^	Microbial log reduction^*c*^
Coarse	0	1.2 ± 0.4	0.23	>6.52
	14	9.6 ± 3.0	0.62	>6.52

Fine	0	0.5 ± 0.1	0.08	>6.52
	14	0.6 ± 0.1	0.08	>6.52

a The coarse emulsion was prepared by adding distilled water to the acidified oil with PGPR under continuous stirring at 700 rpm for 30 min. The fine emulsion was prepared by homogenizing the coarse emulsion by microfluidization at 12 82.7 MPa for 2 passes.

b PDI, polydispersity index.

c Microbial log reduction is calculated as log *N*_0_ − log *N*. Treatments were performed with a 30-min contact time at 22°C against Salmonella Enteritidis desiccated to 75% ERH. The inoculum level was 7 log CFU, and the detection limit was 0.48 log MPN per stainless steel coupon. When bacterial survival was reduced to a level below the detectable limit, the result was interpreted as a reduction of >6.52 log MPN/coupon.

### Water concentration and water activity.

**FIG 3:**
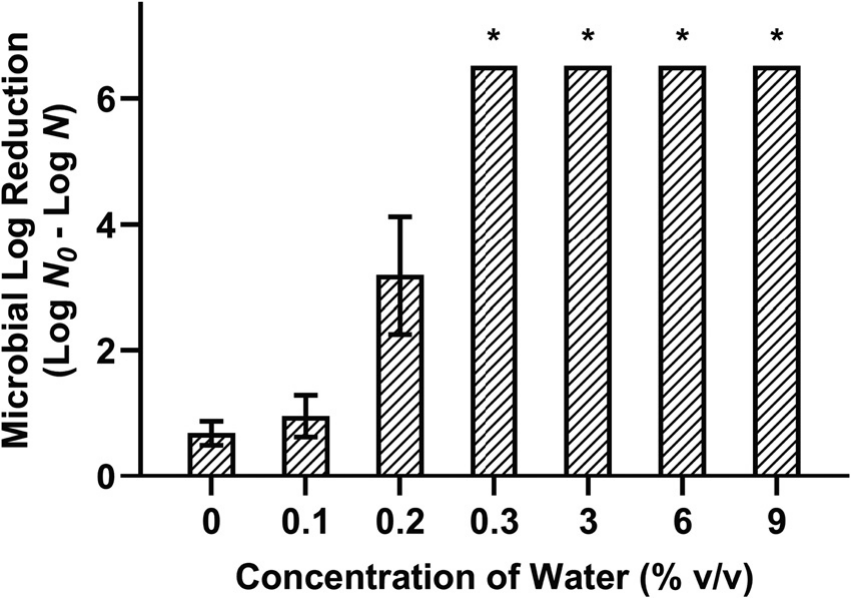
Influence of the water concentration on the efficacy of acidified W/O emulsions against desiccated Salmonella Enteritidis phage type 30. All treatment formulations were tested against cells desiccated to 75% ERH with a 30-min treatment time at 22°C with 200 mM acetic acid and various water concentrations in the acidified W/O emulsions. The inoculum level was 7 log CFU per stainless steel coupon, and the detection limit was 0.48 log MPN. *, bacterial survival was reduced to below the detectable limit, i.e., a reduction of >6.52 log MPN/coupon.

The antimicrobial efficacy of the acidified W/O emulsion was further assessed as a function of the water concentration and *a_w_*. A 30-min treatment at 22°C with 200 mM acetic acid was tested against cells desiccated to 75% ERH. Dispersing a low concentration of water (0.3%, vol/vol) within the acidified oil with PGPR was sufficient to achieve the enhancement, reducing desiccated S. Enteritidis by >6.52 log MPN/coupon (Fig. 3). However, the changes in the water concentration were accompanied by changes in the *a_w_* of the solution (Table 2). The measured *a_w_* value of acidified oil (no water) was 0.33. The *a_w_* values of acidified W/O emulsions formulated with 0.1%, 0.2%, and 0.3% water were 0.64, 0.67, and 0.78, respectively. These results indicated that the water concentration may not be the sole factor in the enhanced efficacy of the acidified W/O emulsion.

**TABLE 2:** Measured water activity of acidified W/O emulsions with various water concentrations^*a*^

Water concn (%, vol/vol)	*a_w_*
0	0.33
0.1	0.64
0.2	0.67
0.3	0.78
1	0.92
2	0.93
3	0.93
6	0.94
9	0.96

a All emulsions had 200 mM acetic acid in the final solution. The solution water activity (*a_w_*) was measured with the dewpoint method at 22°C.

To elucidate the role of *a_w_*, glycerol-water mixtures were used as the emulsion aqueous phase to formulate a series of acidified W/O emulsions with a constant water level (1%) but with various levels of *a_w_* (formulations A to H) (Table 3). Treatment with the acidified W/O emulsion without glycerol (formulation A) (*a_w_* of 0.93) reduced desiccated S. Enteritidis by >6.52 log MPN/coupon. The solution *a_w_* decreased with increasing levels of glycerol. Formulations D to H showed *a_w_* values of 0.60, 0.57, 0.50, 0.46, and 0.28, respectively, which reduced desiccated S. Enteritidis by 3.34, 2.17, 1.51, 0.97, and 0.72 log CFU/coupon, respectively, and reduced desiccated L. monocytogenes by 3.91, 2.16, 1.47, 1.06, and 0.82 log CFU/coupon, respectively. These results showed that the antimicrobial efficacy at a constant water level was reduced as the *a_w_* of the acidified W/O emulsion was reduced. Thus, the enhanced efficacy of the acidified W/O emulsion was likely due to osmotic pressure. We acknowledge that since the surfactant PGPR was prepared at 3% (wt/wt) in oil, its final concentration in the acidified W/O emulsion would decrease with increasing aqueous-phase concentrations. However, as surfactants were not found to influence the treatment efficacy, we believe that it was not an issue in this experiment.

**TABLE 3:** Influence of water activity on the antimicrobial efficacy of acidified W/O emulsions with a constant water concentration (1%, vol/vol)

Formulation (*V*_water_:*V*_glycerol_)^*a*^	Final concn (%, vol/vol) in acidified W/O emulsion^*b*^		*a_w_* ^*c*^	Mean microbial log reduction ± SD^*d*^	
	Water	Glycerol		Salmonella Enteritidis phage type 30	Listeria monocytogenes LM25
A (1.00:0.00)	1	0	0.93	>6.52	>6.52
B (0.75:0.25)	1	0.33	0.76	>6.52	>6.52
C (0.50:0.50)	1	1	0.67	>6.52	>6.52
D (0.45:0.55)	1	1.22	0.60	3.34 ± 0.28	3.91 ± 0.46
E (0.40:0.60)	1	1.50	0.57	2.17 ± 0.59	2.16 ± 0.39
F (0.30:0.70)	1	2.33	0.50	1.51 ± 0.22	1.47 ± 0.10
G (0.25:0.75)	1	3	0.46	0.97 ± 0.13	1.06 ± 0.11
H (0.10:0.90)	1	9	0.28	0.72 ± 0.05	0.82 ± 0.07

a *V*, volume fraction. The volume fractions of water and glycerol in the aqueous phase of the acidified W/O emulsion were defined as *V*_water(or glycerol)_/*V*_water+glycerol_.

b All emulsions had 200 mM acetic acid in the final solution.

c The solution water activity (*a_w_*) was measured with a dewpoint *a_w_* meter at 22°C.

d Microbial log reduction was calculated as log *N*_0_ − log *N*. Treatments were performed with a 30-min contact time at 22°C against cells desiccated to 75% ERH. The inoculum level was 7 log CFU, and the detection limit was 0.48 log MPN per stainless steel coupon. When bacterial survival was reduced to a level below the detectable limit, the result was interpreted as a reduction of >6.52 log MPN/coupon.

### Antimicrobial resistance of cells with different levels of desiccation.

The influence of cellular dehydration on antimicrobial sensitivity was explored by desiccating cells to different ERH levels before treatment. For the treatments with oil, nonacidified W/O emulsions, and acidified oil, greater resistance of both S. Enteritidis and L. monocytogenes was observed in cells desiccated to 33% ERH compared to those desiccated to 75% ERH (Table 4). When the acidified W/O emulsion was used for treatment, MLRs of >6.52 log MPN/coupon were observed for both desiccated bacteria regardless of the desiccation levels and temperatures (Table 4).

**TABLE 4:** Influence of the desiccation level on the resistance of Salmonella Enteritidis phage type 30 and Listeria monocytogenes LM25 to treatment with acidified oil and acidified W/O emulsions at different temperatures

Formulation and treatment temp (°C)	Inoculum desiccation level (ERH) (%)	Mean microbial log reduction ± SD^*a*^	
		Salmonella Enteritidis phage type 30	Listeria monocytogenes LM25
Oil			
22	75	X	X
22	33	X	X
45	75	0.35 ± 0.05	0.47 ± 0.07
45	33	X	0.19 ± 0.06^*b*^
W/O emulsion (3% [vol/vol] water, 3% [wt/wt] PGPR)			
22	75	X	X
22	33	0.16 ± 0.02	0.13 ± 0.05
45	75	0.91 ± 0.08	0.33 ± 0.12
45	33	0.59 ± 0.07^*b*^	0.14 ± 0.04^*b*^
Acidified oil (200 mM acetic acid)			
22	75	0.74 ± 0.06	1.43 ± 0.33
22	33	0.05 ± 0.03^*b*^	0.38 ± 0.08^*b*^
45	75	1.40 ± 0.05	1.92 ± 0.45
45	33	0.11 ± 0.06^*b*^	0.83 ± 0.15^*b*^
Acidified W/O emulsion (200 mM acetic acid, 3% [vol/vol] water, 3% [wt/wt] PGPR)			
22	75	>6.52	>6.52
22	33	>6.52	>6.52
45	75	>6.52	>6.52
45	33	>6.52	>6.52

a Microbial log reduction was calculated as log *N*_0_ − log *N*. Treatments were performed with a 30-min contact time at the indicated temperatures. The inoculum level was 7 log CFU and was desiccated to the indicated ERH. The limit of detection was 0.48 log MPN per stainless steel coupon. When bacterial survival was reduced to a level below the detectable limit, the result was interpreted as a reduction of >6.52 log MPN/coupon. X, no MLR was observed.

b For the same organism, formulation, and treatment temperature, the MLR difference between the cells desiccated to 75% ERH and those desiccated to 33% ERH was statistically significant (*P *< 0.05). A comparison was not drawn when there was no MLR.

**FIG 4:**
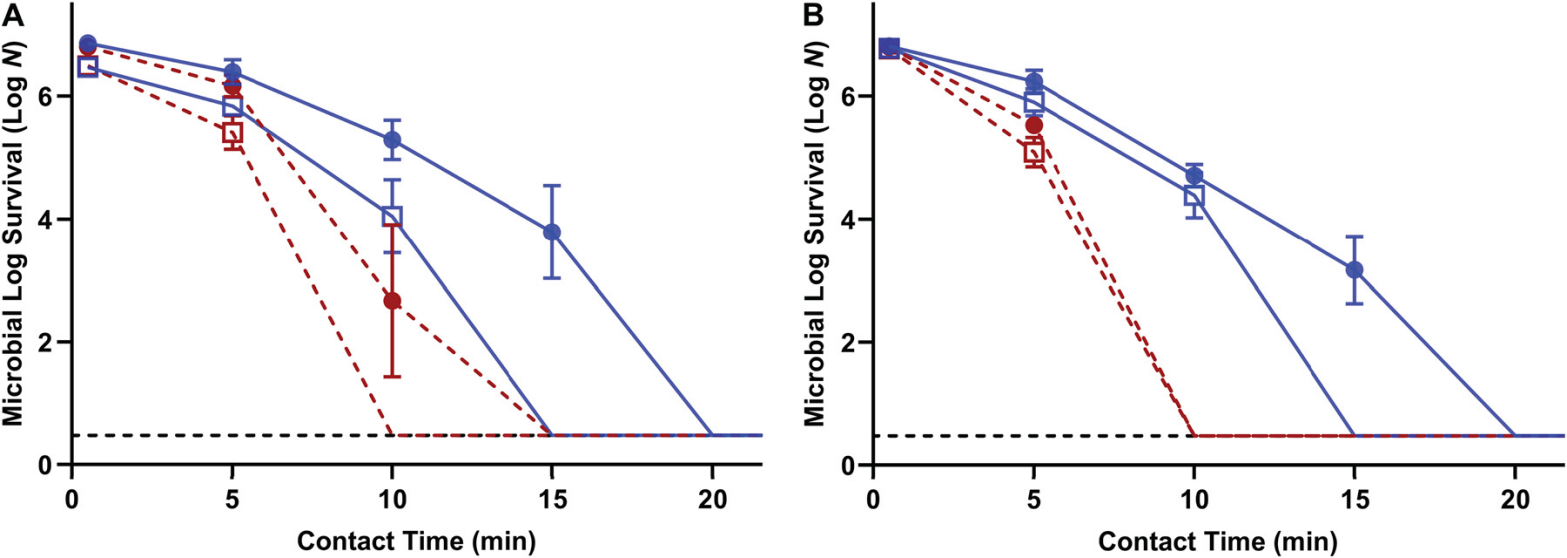
Influence of the desiccation level and temperature on the survival of Salmonella Enteritidis phage type 30 (A) and Listeria monocytogenes LM25 (B) when treated with acidified W/O emulsions over time. Cells desiccated to 75% ERH (closed circles) and 33% ERH (open squares) were treated with acidified W/O emulsions (200 mM acetic acid, 3% [wt/wt] PGPR, 3% [vol/vol] distilled water) at 22°C (solid blue lines) or 45°C (dashed red lines). The inoculum level was 7 log CFU per stainless steel coupon, and the detection limit was 0.48 log MPN. The initial time point was 30 s. Once samples reached levels that were below the detection limit (dotted black lines) and remained there upon subsequent incubation, the points were omitted from the graph.

**FIG 5:**
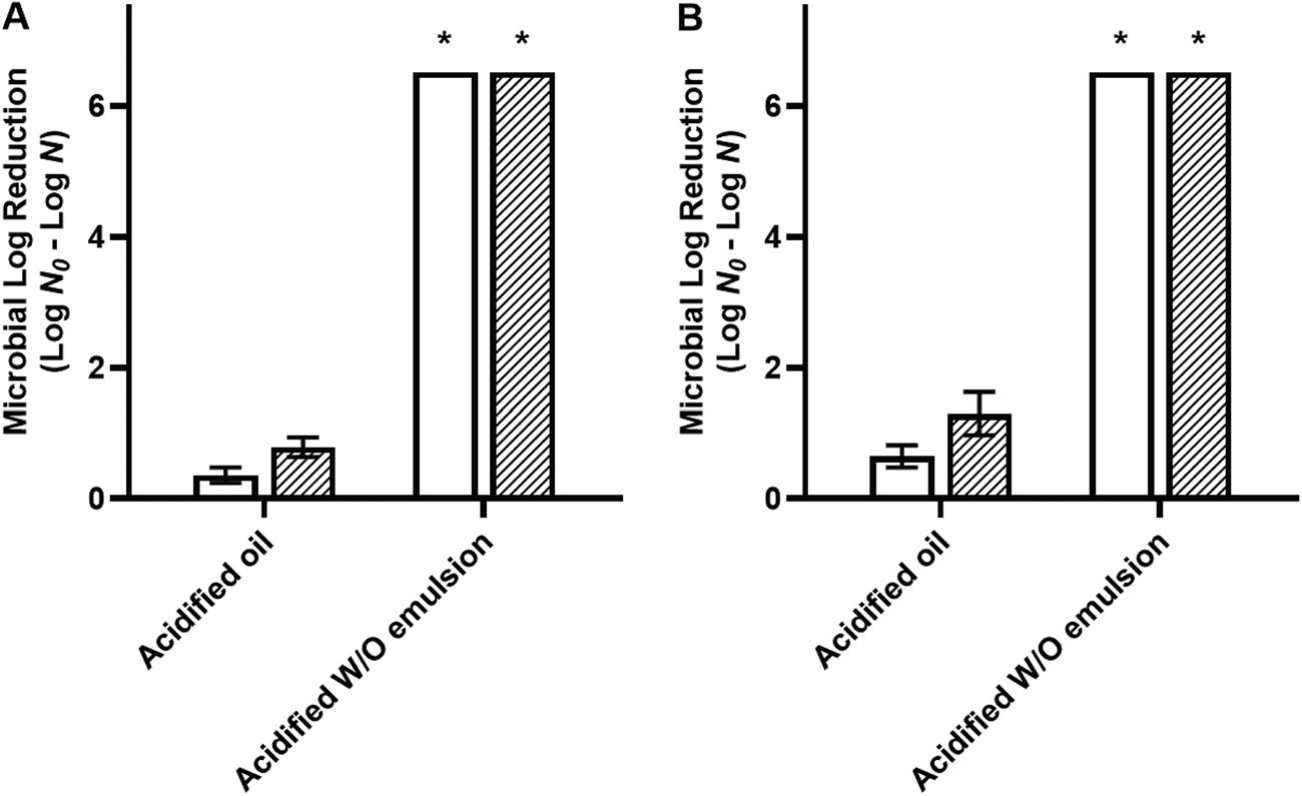
Efficacy of acidified oil and acidified W/O emulsions with a 20-min treatment at 22°C (A) and a 15-min treatment at 45°C (B) against desiccated cocktails of Salmonella (filled bars) and Listeria monocytogenes (striped bars). Cells desiccated to 75% ERH were treated with acidified oil and acidified W/O emulsions with 200 mM acetic acid. The aqueous phase in acidified W/O emulsions was 3% (vol/vol) distilled water. The inoculum level was 7 log CFU per stainless steel coupon, and the detection limit was 0.48 log MPN. *, bacterial survival was reduced to below the detectable limit, i.e., a reduction of >6.52 log MPN/coupon.

As both desiccated bacteria were reduced to levels below the detectable limit by a 30-min treatment with acidified W/O emulsions, a time course study was further conducted (Fig. 4). Increased treatment efficiency was observed when the acidified W/O emulsion was tested against cells desiccated to 33% ERH compared to those desiccated to 75% ERH. A minimal contact time of 20 min at 22°C or 15 min at 45°C was required to reduce desiccated S. Enteritidis phage type 30 and L. monocytogenes LM25 by >6.52 log MPN/coupon. Based on these results, the acidified W/O emulsion treatment times (20 min at 22°C and 15 min at 45°C) were selected for bacterial cocktail testing (Fig. 5). The results showed that Salmonella (four-strain cocktail) and L. monocytogenes (three-strain cocktail) cells desiccated to 75% ERH were reduced by >6.52 log MPN/coupon after treatment with acidified W/O emulsions for 20 min at 22°C or 15 min at 45°C.

### Bacterial morphology.

**FIG 6:**
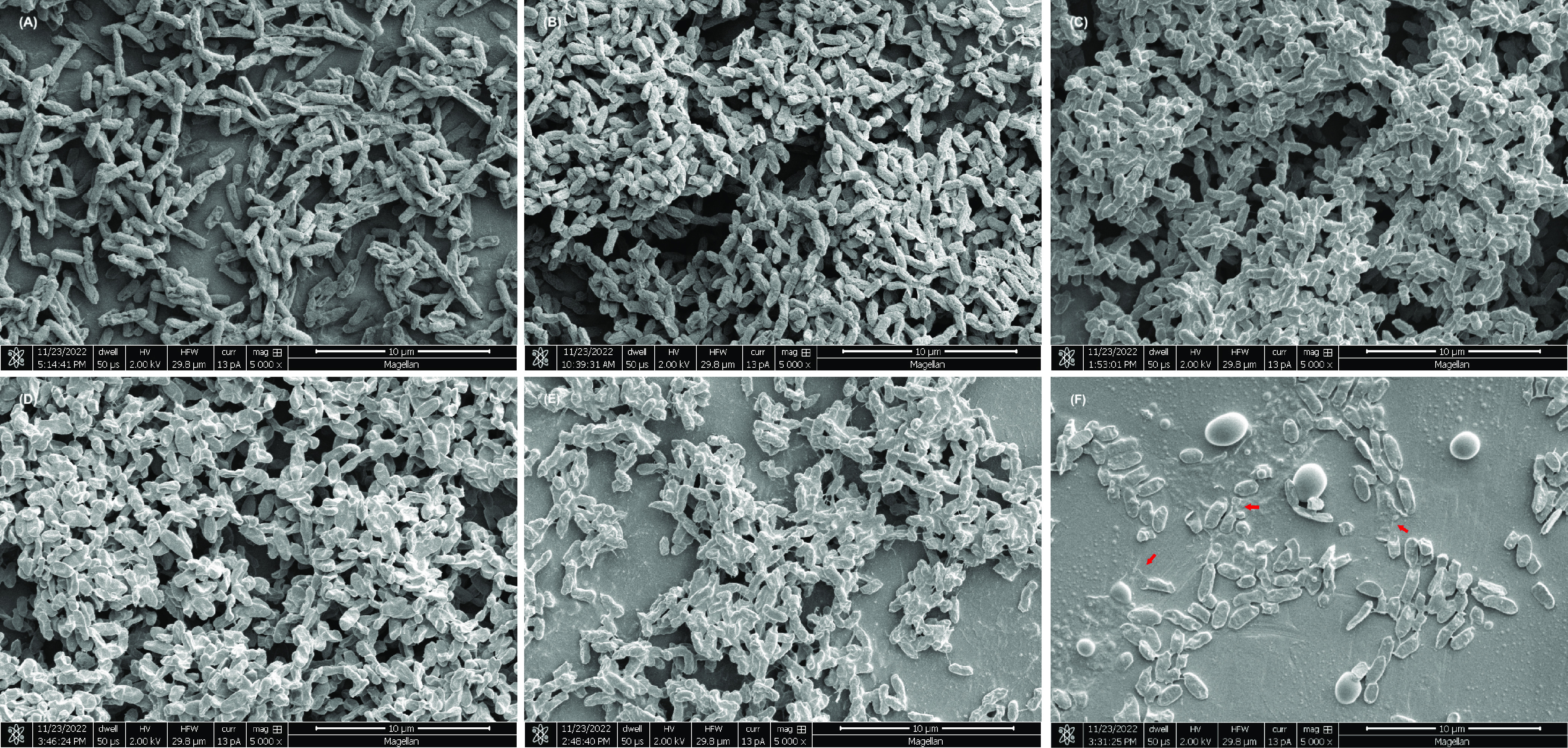
Electron micrographs of Salmonella Enteritidis phage type 30 without and with treatments. Images are as follows: nondesiccated cells (grown on agar plates) without treatment (A), desiccated cells (33% ERH for 20 h) without treatment (B), desiccated cells treated with peanut oil with 3% (wt/wt) PGPR (C), desiccated cells treated with a nonacidified W/O emulsion (3% [vol/vol] distilled water, 3% [wt/wt] PGPR) (D), desiccated cells treated with acidified oil (200 mM acetic acid, 3% [wt/wt] PGPR) (E), and desiccated cells treated with an acidified W/O emulsion (200 mM acetic acid, 3% [wt/wt] PGPR, 3% [vol/vol] distilled water) (F). Treatment was carried out at 22°C for 30 min. Arrows indicate cellular lysis.

The cellular morphology of S. Enteritidis phage type 30 after treatment was explored using scanning electron microscopy (SEM). Cells grown on solid agar surfaces (Fig. 6A) and desiccated cells (33% ERH for 20 h) (Fig. 6B) showed few morphological differences. No changes in cell density or morphology were observed when desiccated cells were treated with nonacidified oil with PGPR (Fig. 6C) and nonacidified W/O emulsions (Fig. 6D). For desiccated cells treated with acidified oil (Fig. 6E), there was a reduction in the cell density, which was consistent with the corresponding MLR data (acidified oil with 200 mM acetic acid at 22°C for 30 min showed a decrease of 0.74 log CFU/coupon, as shown in Fig. 2A). Treatment with the acidified W/O emulsion (Fig. 6F) resulted in a pronounced cell density reduction, with the few remaining cells showing a flattened and/or ruptured morphology, likely indicative of lysis. The cellular damage and the reduction in the cell density are consistent with the corresponding MLR data (the acidified W/O emulsion with 200 mM acetic acid at 22°C for 30 min showed a reduction of >6.52 log MPN/coupon, as shown in Fig. 2A). In addition, the observed round/oval structures were consistent in size with the coarse emulsion droplets, which were likely the residual acidified W/O emulsion treatments.

## DISCUSSION

The environmental water level and osmotic stress have been linked to the antimicrobial activity of organic acids (23–25, 30, 31). Those studies reported an increase in the antimicrobial activity of organic acids with decreasing environmental osmolarity (i.e., an osmotic downshift), such as when bacterial cells were osmotically stressed by a prewash with salt solutions or when cells were desiccated before exposure to organic acids. One proposed mechanism described previously was that the diffusion of acetic acid into the cytoplasm may be retarded due to an increase in membrane rigidity under hypertonic conditions (30). In this study, based on the moisture content of peanut butter, 3% water was dispersed within acidified oil to make acidified W/O emulsions. This addition of water enhanced the treatment efficacy dramatically. Subsequent experiments showed that a minimal water concentration of 0.3% (vol/vol) was sufficient to achieve the enhancement. This water level is 10-fold lower than the water levels in ethanol or isopropanol sanitizers used for dry cleaning, often used at 70% alcohol and 30% water. Although the addition of water to oil-based systems increased the final solution *a_w_*, the acidified W/O emulsion with 0.3% water had an *a_w_* of <0.8, which may be acceptable for adaptation in LMF-processing environments as this level of available moisture was insufficient to support the growth of bacteria and fungi. The *a_w_* of peanut oil can decrease exponentially with increasing temperatures depending on the level of water saturation (32). Thus, the original moisture content of oil samples should be taken into account when adding/dispersing the determined minimum effective concentration of water (0.3%, vol/vol) into acidified oil for antimicrobial enhancement (MLR of >6.52). However, the variation in the *a_w_* of acidified W/O emulsions upon heating was expected to be minor when 3% water was used for making the emulsions, a level far above the water saturation point in oil.

Within a colloidal system such as W/O emulsions, the location of a solute is a dynamic motion where the molecule may leave the existing phase and partition into the other depending on its preferential solubility within the two-phase system (33). Dissolving acetic acid in the water or oil phase prior to making acidified W/O emulsions, intriguingly, did not influence the resulting efficacy (measured with a 30-min treatment at 22°C with 200 mM acetic acid against cells desiccated to 75% ERH) (results not shown). This may be explained by the partitioning of acetic acid within the oil-water system. The partition coefficient (*K*_ow_) is an estimate calculated from the equilibrium mole fractions of a compound in a mixture of two immiscible solvents, usually octanol and water. The logarithm with base 10 of *K*_ow_ (log *P*) ranges from −3 to 10, denoting the affinity of a compound from hydrophilic to lipophilic. Theoretically, a compound with a log *P* value of zero would partition equally between the two immiscible phases. With a log *P* value of −0.17, acetic acid is approximately 10 times more soluble in water than in oil (34). According to the Henderson-Hasselbalch equation, the extent of acid ionization is a function of the environmental pH and the acid dissociation constant (*K_a_*). With a p*K_a_* of 4.75, approximately 99% of the acetic acid remained undissociated at pH 2.7, the measured pH of the acidified W/O emulsion with 200 mM acetic acid (see Fig. S2 in the supplemental material). In the undissociated form, organic acids can permeate the cell membrane until equilibrium is reached, followed by dissociation in the neutral cytoplasm (35). Physiological dysfunctions were subsequently induced by the cytoplasmic accumulation of protons and anions, e.g., intracellular acidification, metabolic disruption, membrane potential disruption, and perturbation of osmolyte pools, among other detrimental impacts (36). Our previous work investigated the influence of acetic acid-acidified oil on the structural integrity of S. Enteritidis phage type 30 using transmission electron microscopy (TEM) (25). Membrane disruption, periplasmic irregularity, as well as cell lysis were revealed. Granularity in the cytoplasm was also observed, possibly indicating protein aggregation in the cytoplasm as a response to the acidified-oil stress.

Osmosis describes the movement of water from low- to high-osmolarity regions, and the pressure created by such water flux is called osmotic pressure. According to the Van’t Hoff equation, the osmotic pressure of a solution is positively correlated with its solute concentration and inversely related to its *a_w_* (37). At equilibrium vapor pressure, the *a_w_* within the environment can be measured with ERH using the equation ERH = *a_w_* × 100%. In the case of bacterial desiccation, the loss of moisture would lead to an increase in cytoplasmic osmolarity. Thus, hypoosmotic stress would be formed when desiccated cells were subjected to environments with lower osmolarity (i.e., higher *a_w_*). Glycerol is a common humectant commonly used to decrease the *a_w_* of foods due to its strong attraction force with water and is naturally produced by fungi to combat dry environments (38). Nakagawa and Oyama characterized the physical and chemical properties of water-glycerol mixtures on a molecular basis (39). Those authors classified the state of water within such a mixture into three *a_w_* regions, further ascribed to the molecular interactions with different water-glycerol fractions. Most water interacted with glycerol via hydrogen binding at an *a_w_* of <0.7 and was present as small, isolated clusters in the solution. Above this point, excessive water was separated from the water-glycerol mixtures and exhibited bulk-like behavior, namely, intermediate water. At an *a_w_* of >0.8, bulk water behavior was dominant due to the faster molecular dynamics in the solution, meaning that the water molecules at this state were available for use. Our results showed that the addition of glycerol to the water phase of the acidified W/O emulsion reduced the solution *a_w_*, and the efficacy of the acidified W/O emulsion was attenuated. This indicated that osmotic pressure is involved in microbial killing by the acidified W/O emulsion. The time course study (Fig. 4) also supported that the acidified W/O emulsion was more efficient against cells desiccated to 33% ERH than the less-dried ones. Hypoosmotic stress alone (nonacidified W/O emulsion) was not antimicrobial, and acetic acid stress alone (acidified oil without water droplets) showed low levels of efficacy against desiccated bacteria. However, combining the two stresses (acidified W/O emulsion) produced synergistic MLRs (Table 4). Due to unbalanced vapor pressure, the water molecules within the cells can diffuse through the cell membrane to a contacted oil phase until equilibrium (40). In agreement with this, our system illustrated that between desiccated cells and acidified W/O emulsions, water influx can occur upon an osmotic downshift, possibly lysing the cells with damaged membranes (25).

There may be alternative explanations for the modes of action of acidified W/O emulsions. When there is an imbalance between intracellular and extracellular fluid osmolarity, cells can respond via a series of mechanisms known as osmoadaptation (41). Membrane proteins such as osmosensing (OS) transporters and mechanosensitive (MS) channels are responsible for osmosensing and osmoregulation (42, 43). Solute uptake via OS transporters is activated upon osmotic upshifts, and the MS channels open upon osmotic downshifts to allow solute efflux, attenuating water influx. A previous study utilizing Escherichia coli mutants with MS channels knocked out reported that the mutants showed lower survival rates than the wild type upon rapid osmotic downshifts (44). Thus, damage to the osmoregulatory proteins could also be involved in the antimicrobial action of acidified W/O emulsions, leading to a loss of turgor and cell burst upon osmotic downshifts. Alternatively, the water in the acidified W/O emulsion may act as a catalyst promoting acetic acid-induced protein denaturation. This proposed mechanism is based on an example where pure ethanol was found to be less bactericidal than diluted ethanol as the presence of water facilitated protein denaturation (45). In addition, alcohol has been found to exhibit evaporation profiles as a pure compound that are different from those as a mixture with water (46, 47). Instead of completely evaporating before water, a proportion of the alcohol remained in the binary mixture until all liquid had evaporated. Thus, the rate at which alcohol evaporated was reduced by the introduction of water, allowing increased contact within a specific holding duration.

Grasso et al. validated the alcohol-based sanitization method used commercially for peanut butter processing (21). In this context, Salmonella-inoculated peanut butter was used to contaminate a pilot-scale processing line. For cleaning, a rinse with cold oil (22°C) was used for flushing out large clumps, followed by another rinse with warm oil (60°C) to remove residual debris. When the discharge cleared, the piping was circulated with hot oil (93°C for 2 h) as a thermal treatment. Next, the processing line was cooled for 24 h to ensure that subsequent sanitization with 60% isopropyl alcohol (22°C for 1 h) was carried out at temperatures below its flash point (40°C). The initial contamination level was 7.4 log CFU/g of peanut butter. After the cold-oil rinse, the count was 6.3 log CFU/g as sampled from the discharge. After the hot-oil treatment, the count was 3.2 log CFU/g as sampled from the discharge. The numbers of surviving cells remaining in the processing line were approximately 1 log CFU/cm^2^, as determined by swabbing the surfaces at high risk of fouling. After alcohol-based sanitization, that level was reduced to below the detection limit of 0.16 log CFU/cm^2^.

Compared to the alcohol-based method, the application of antimicrobial oils for sanitization would preclude the need for a system cooldown as oils are not flammable at the preceding cleaning temperatures, preventing production downtime. Desiccated Salmonella (4-strain cocktail) and L. monocytogenes (3-strain cocktail) were reduced by >6.52 log MPN/coupon after treatment with acidified W/O emulsions at 22°C for 20 min with 200 mM acetic acid. This study has shown that acidified W/O emulsions could be highly effective sanitizing treatments for LMF-processing equipment.

## MATERIALS AND METHODS

### Bacterial strains and inoculum preparation.

Salmonella enterica subsp. *enterica* serovar Enteritidis phage type 30 (ATCC BAA-1045) (outbreak strain associated with almonds) and three other serovars recommended for testing the efficacy of produce sanitizers (48) (*S*. Michigan ATCC BAA-709, *S*. Montevideo ATCC BAA-710, and *S*. Gaminara ATCC BAA-711) were obtained from the American Type Culture Collection (ATCC) (Manassas, VA). Strains of Listeria monocytogenes were initially obtained from Cornell University and randomly designated LM7 to LM37 when received at the University of Massachusetts—Amherst (49). Three strains of different genetic lineages (lineage I, LM25; lineage II, LM28; lineage III, LM23), sourced from human sporadic and epidemic cases, were used in this study. Stock cultures were stored at −80°C in tryptic soy broth (TSB; Difco, Becton, Dickinson, Sparks, MD) with 25% glycerol (catalog number G7893; Sigma-Aldrich, St. Louis, MO). Working cultures were prepared by streaking stock cultures onto tryptic soy agar (TSA; Difco, Becton, Dickinson) with incubation overnight at 37°C, which were maintained at 4°C and replaced monthly.

The inoculum was prepared according to a procedure described previously by Uesugi et al. (50). Before each experiment, an isolated colony was transferred from the working culture to 20 mL of TSB and incubated at 37°C for 24 h. Subsequently, 100 μL of this liquid culture was spread onto TSA with 0.6% yeast extract (TSAYE), with incubation at 37°C for 24 h to produce bacterial lawns. These sessile cells were harvested using sterile scrapers (Fisher Scientific, Pittsburgh, PA) and resuspended in distilled water (18.2 MΩ · cm) (Direct-Q water purification system; Merck KGaA, Darmstadt, Germany) as the inoculum. The optical density at 600 nm (OD_600_) values were adjusted to 1.2 for Salmonella strains and 1.5 for L. monocytogenes strains to achieve approximately 10^9^ CFU/mL. The inoculum was diluted with 0.1% peptone water (Difco, Becton, Dickinson) and plated onto TSAYE to confirm cell numbers. For cocktail studies, this procedure was repeated with each strain, and the strains were combined to make a four-strain Salmonella cocktail or a three-strain L. monocytogenes cocktail.

### Desiccation of bacteria.

An aliquot (20 μL) containing approximately 10^7^ CFU of cells was added to a stainless steel coupon (2B-finish; Biosurface, Bozeman, MT) and held in a desiccator at room temperature (20°C to 22°C) for 20 h. Saturated solutions of magnesium chloride (catalog number 7786-30-3; Sigma-Aldrich) and sodium chloride (catalog number S271-1; Fisher Scientific) were used to maintain the environment at 33% and 75% equilibrium relative humidity (ERH), respectively, as indicated by a hygrometer placed within the desiccator. The levels of desiccation were selected for antimicrobial assays based on a range of ERH levels that can occur at processing facilities for LMFs such as peanut butter and chocolate. The desiccation time was selected based on the results of a previous study by Gruzdev et al., which showed that cellular dehydration reached a maximum level after 20 h of desiccation (23). Used coupons were soaked in acetone overnight to degrease them, washed with distilled water, autoclaved, and dried for reuse (25).

### Acidified oil and W/O emulsions.

Unless otherwise specified, peanut oil was used as the carrier oil within the tested systems. Other types of oil were also tested throughout experimentation. These included mineral oil, long-chain triglycerides (LCTs) such as soybean and corn oil, and medium-chain triglycerides (MCTs). Lipophilic surfactants were dissolved in the carrier oil with continuous stirring at 400 rpm for 30 min at room temperature. An excessive surfactant concentration, 3% (wt/wt) in oil, was used to prevent droplet coalescence. Polyglycerol polyricinoleate (PGPR 4150; Palsgaard, Juelsminde, Denmark) was used as the surfactant within the tested systems unless otherwise specified. Sorbitan monooleate (Span 80; Sigma-Aldrich) was also tested. Glacial acetic acid (catalog number A38-500; Fisher Scientific) was dissolved in oil or the oil-surfactant mixture at the desired concentrations to create acidified oil.

To prepare acidified W/O emulsions, distilled water was added to the acidified oil with the surfactant under continuous stirring at 700 rpm for 30 min at room temperature. This spontaneous emulsification method (i.e., coarse) was used within the tested systems unless otherwise specified. Fine emulsions were also tested throughout experimentation, as prepared by pumping coarse emulsions through a microfluidizer at 82.7 MPa for 2 passes (catalog number M-110L; Microfluidics, Newton, MA). Emulsion droplet/particle sizes (intensity-weighted mean diameter, Z average) and distributions (polydispersity index [PDI]) were measured by dynamic light scattering (DLS) using the Zetasizer Nano ZS system (Malvern Instruments, Worcestershire, UK). Before DLS analysis, W/O emulsions were diluted with hexadecane (refractive index = 1.434; viscosity = 3.13 mPa · s at 22°C) at a ratio of 1:100 to prevent multiscattering (29).

### (i) Measurement of pH.

To measure the pH of acidified W/O emulsions, the electrode of a potentiometric pH meter (FiveEasy F20; Mettler-Toledo, Greifensee, Switzerland) was dipped into diluted emulsions with continuous stirring to allow the proper flow of ion exchange between emulsion droplets and the electrode solution. Dilutions were made in distilled water (pH 5.8), where the proportion of the original emulsions did not exceed 50% (vol/vol), to prevent damaging the porosity of the electrode glass membrane (51).

### (ii) Osmotic pressure assay.

Glycerol was added to the aqueous phase of W/O emulsions to adjust the solution *a_w_*. The emulsion aqueous phase was formulated using a series of glycerol-water fractions (39). This allowed us to investigate the influence of osmotic pressure on the antimicrobial efficacy of acidified W/O emulsions with a constant water level. The dewpoint method was used to determine the solution *a_w_* using the AquaLab series 3 system (v2.3; Meter Group, Pullman, WA) in continuous mode at 22°C. The mean value was reported when two consecutive measurements fell within a range of ±0.002 (52).

### Antimicrobial assay.

A treatment solution (100 μL) was dispensed onto the desiccated bacteria on a stainless steel coupon, which was subsequently transferred to an incubator set at a constant temperature for holding for up to 30 min. For temperature studies, the solutions were preadjusted to 22°C or 45°C. The come-up time during treatment for a stainless steel coupon (12.7-mm diameter by 3.8-mm thickness) to reach 45°C from room temperature was expected to be short and therefore was not measured. At the end of the treatment, one stainless steel coupon was transferred to 10 mL of TSB in a conical polypropylene tube with sterile glass beads (diameter, 1 mm) and vortexed at 3,200 rpm for 2 min to ensure the removal of cells from the coupon into the medium. The addition of a treatment solution with 500 mM acetic acid (100 μL) decreased the pH of TSB (10 mL) from 7.30 to 6.81. Microbial survival was determined by plating cells onto TSAYE. Enumeration was performed after incubation of the plates at 37°C for 24 h. A longer incubation time (48 h) did not produce different colony counts. Thus, the 24-h incubation time was used for all assays. In cases where bacterial survival was reduced to a level below the detection limit of 2 log CFU/coupon with the plate count method, the experiments were repeated, and the most probable number (MPN) was reported.

The MPN was determined according to methods in the *Bacteriological Analytical Manual* (BAM) of the U.S. Food and Drug Administration (53). In this context, transferring one stainless steel coupon into 10 mL of TSB upon the end of treatment was considered a 1:10 dilution. From here, aliquots of 1, 0.1, and 0.01 mL with three tubes each were added to 20 mL of TSB per tube. This allowed MPN determination per stainless steel coupon to match the MPN per milliliter on the BAM table reference where inocula of 0.1, 0.01, and 0.001 mL were used. Growth was indicated by turbidity after incubation at 37°C for 24 h. Confirmatory tests were performed by plating the turbid cultures on selective medium at 37°C for 24 h. Xylose lysine deoxycholate (XLD) agar (catalog number R459902; Fisher Scientific) was used to confirm Salmonella. Oxford agar (catalog number CM0856B; Fisher Scientific) supplemented with *Listeria* selective agent (catalog number SR0206; Fisher Scientific) was used to verify L. monocytogenes. When all three tubes at each dilution were negative (0, 0, and 0), the outcome was interpreted as less than the outcome with negative tubes at the two lowest dilutions and only one positive tube at the highest dilution (0, 0, and 1). Thus, the detection limit was 3 MPN/coupon (0.48 log MPN/coupon).

Microbial log reduction (MLR) was calculated with base 10 as follows: MLR = log(*N*_0_/*N*) = log *N*_0_ − log *N*, where *N*_0_ is the viable count of desiccated cells recovered from stainless steel coupons and *N* is the CFU or MPN of surviving cells after treatment. When *N*_0_ equals *N*, the treatment is interpreted as being not antimicrobial.

### Scanning electron microscopy.

Bacteria were desiccated on stainless steel coupons and treated with antimicrobial formulations as described above. If bacteria were treated, sterile peanut oil was used to rinse off the treatment solutions from the coupons. Controls included untreated desiccated and untreated nondesiccated cells, prepared by adding sessile cells to coupons and drying the coupons briefly in a biosafety cabinet at room temperature for 15 min before fixation.

### (i) Sample preparation.

The preparation of biological specimens for scanning electron microscopy (SEM) observation was carried out at room temperature according to a procedure described previously by Fischer et al. (54). Treatment and control coupons were fixed with 2.5% glutaraldehyde (catalog number G6257; Sigma-Aldrich) in 0.2 M sodium cacodylate (pH 7.4) (catalog number C0250; Sigma-Aldrich) for 30 min, rinsed with 0.2 M sodium cacodylate, postfixed with 2% osmium tetroxide (OsO_4_) (catalog number 75632; Sigma-Aldrich) in 0.2 M sodium cacodylate for 30 min, and rinsed again with 0.2 M sodium cacodylate. Subsequently, the fixed samples were dehydrated with a graded ethanol series (30 to 100% at 10% intervals for 10 min each) and postdried with hexamethyldisilazane (HMDS) (catalog number 440191; Sigma-Aldrich) for 20 min. The dried samples were mounted onto SEM specimen stubs using conductive double-sided carbon tapes (catalog number 77825-12; Electron Microscopy Sciences, Hatfield, PA) and coated with 10 nm of gold (108auto sputter coater; Cressington Scientific Instruments, UK). Electron micrographs were obtained at an acceleration voltage of 2 kV using a Magellan 400 XHR-SEM instrument (FEI Company, Hillsboro, OR) at the Electron Microscopy Facility, University of Massachusetts—Amherst.

### Statistical analysis.

Experiments were conducted in three replicates independently. Results were expressed as means ± standard deviations. Differences were determined to be statistically significant at a *P* value of <0.05. Paired *t* tests were performed with data obtained from subjecting the same organism to two different conditions. Unpaired *t* tests were performed with data obtained from subjecting two different organisms to the same condition. Two-way analysis of variance (ANOVA) with Tukey’s *post hoc* test was performed using GraphPad Prism 9 (GraphPad Software, San Diego, CA) to determine the levels of statistical significance.
