# Supplementary material for: Oil-Based Sanitization in Low-Moisture Environments: Delivery of Acetic Acid with Water-in-Oil Emulsions
Source: Microbiol Spectr. 2023 Apr 5;11(3):e05293-22. doi: 10.1128/spectrum.05293-22 (PMC10269857; doi:10.1128/spectrum.05293-22)
Supplement: Supplemental file 1 — Fig. S1 and S2. Download spectrum.05293-22-s0001.pdf, PDF file, 0.5 MB [file spectrum.05293-22-s0001.pdf]

Supplemental Materials

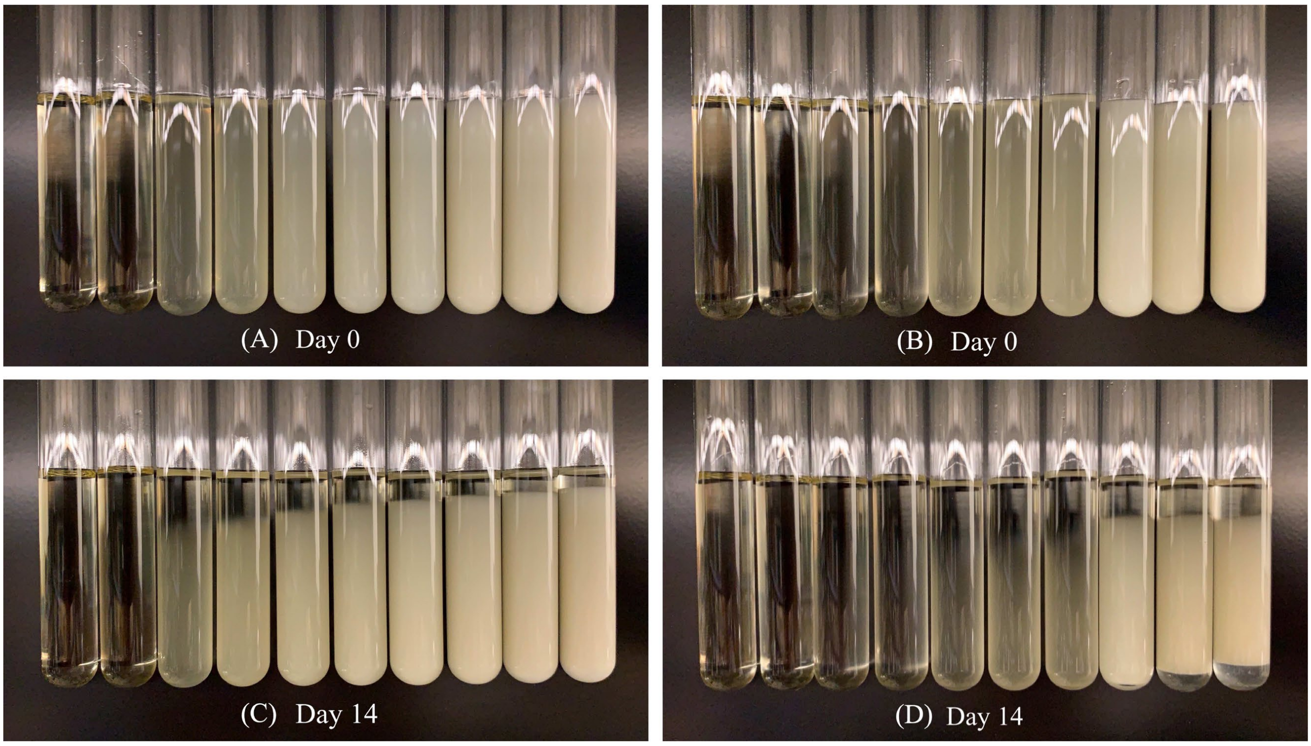

Figure S1. Digital photographs taken upon formation (day 0) and at the end of storage at 22 °C (day 14) of the coarse acidified water-in-peanut oil emulsions stabilized by 3% w/w PGPR (A and C) and Span 80 (B and D) with 200 mM acetic acid and different concentrations of water (left to right: 0%, 0.1%, 0.2%, 0.3%, 0.4%, 0.5%, 0.7%, 1%, 3%, 6%, 9%). The emulsions were prepared by adding distilled water to acidified oil with continuous stirring at 700 rpm for 30 min.

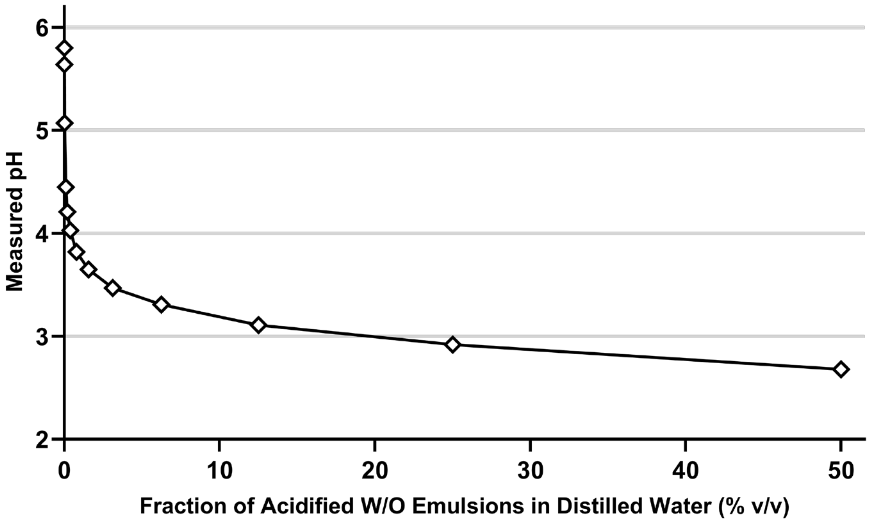

Figure S2. Measured pH of acidified W/O emulsion (200 mM acetic acid), as diluted with distilled water (pH = 5.8).
